# Near visual acuity in an inner city Hispanic community: understanding the barriers and benefits of correction

**Published:** 2014

**Authors:** Thomas Wubben, Gregory Wolfe, Christopher Guerrero, Wanda Jettka Korcz, David J Ramsey

**Affiliations:** MSc/PhD student, College of Medicine, University of Illinois at Chicago, Chicago, USA. twubben1983@yahoo.com; Optometrist: Southern Arizona VA Health Care System, Southern California College of Optometry, Fullerton, USA.; Physician: Department of Family Medicine, University of Illinois at Chicago, Chicago, USA.; Clinical instructor: College of Nursing, University of Illinois at Chicago, Chicago, USA.; Vitreoretinal fellow: Massachusetts Eye and Ear Infirmary, Harvard Medical School, Boston, USA.

Presbyopia is age-related loss of accommodation that gradually impairs near vision. Few studies have examined the burden of presbyopia in the United States of America (USA)^1^,^2^ and none have examined it in economically disadvantaged or minority populations, in which there are increased rates of visual impairment and decreased use of eye care services.^3^,^4^

In our study, individuals ≥35 years of age who attended an employment fair sponsored by the Illinois Department of Employment Services (IDES) in the Pilsen neighborhood of Chicago were invited to undergo testing of their near vision and to befitted with reading spectacles, if needed. Individuals with bilateral visual impairment or blindness, as determined by both a history and a screening examination with a pen light (to exclude gross abnormalities) were given information and instructed to seek an eye examination. In the remaining individuals, near acuity was measured at 40 cm, and 133 people were identified as having functional presbyopia (i.e., near acuity could be improved by at least one line by placing a plus lens in front of either eye).^5^ After this intervention, the 133 individuals with presbyopia were invited to complete a questionnaire based on the National Eye Institute's Visual Function Questionnaire. They were asked about the benefits to them of correcting near vision and about what they considered to be the barriers to having their near vision corrected. All 133 completed the questionnaires.

**‘Employment status correlated with the frequency with which patients accessed an eye care professional’**

**Figure:**
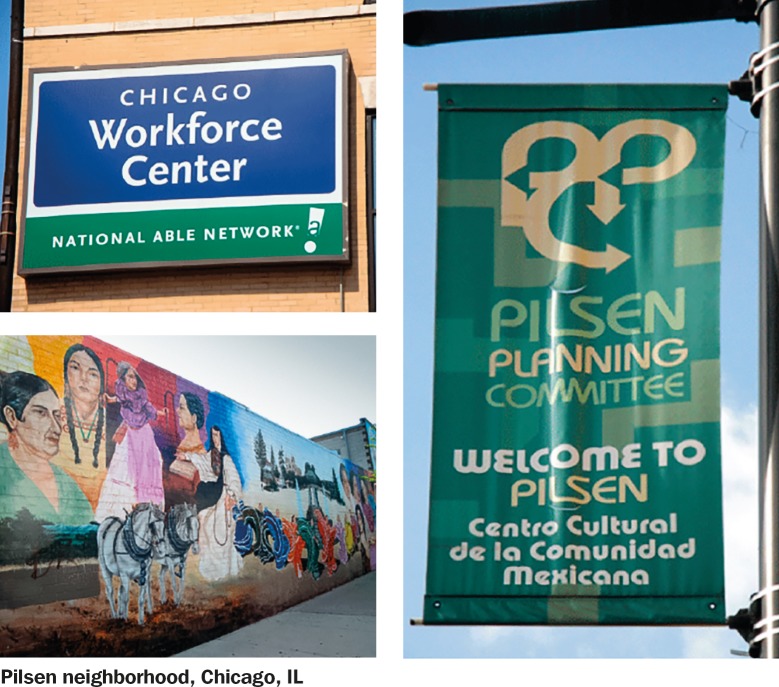
Pilsen neighborhood, Chicago, IL

The 133 respondents ranged in age from 36–85 years old. Their average age was 55 years and 57% were female (74 out of 133). Over 70% (n=96, 50 male participants and 46 female participants) were unemployed. The average uncorrected near acuity was 20/50 (6/15). After receiving reading spectacles, 80% achieved a near acuity of ≥20/25 (6/7.5). Uncorrected near acuity <20/50 (6/15) was associated with greater difficulty reading print (p<0.02) and perceived worse eyesight (p<0.0001) compared to those with uncorrected near visual acuity of ≥20/50.

More than 98 per cent of respondents stated they could read. An improved ability to read was cited as the most important benefit of reading spectacles, with minimal impact perceived on other daily tasks (Table 1). Those who stated that reading spectacles would impact the task of reading print only to some degree or not at all were younger (50±6 years versus 56±9 years: p<0.01) and had better uncorrected near visual acuity (>6/12 versus <6/15: p<0.05). Similar associations were observed between uncorrected near vision and the perceived impact of reading spectacles on other tasks such as preparing meals, using hand tools or sewing, as well as grooming, shaving, or applying makeup. Neither employment status nor gender had a statistically significant impact on the perceived benefits of reading spectacles.

With regards to barriers to obtaining near vision correction, 49 percent (n=64) of the respondents said that the cost of spectacles was a disincentive and 48 per cent (n=63) said that the lack of availability of an eye care professional greatly impeded them from obtaining spectacles (Table 1). Considering that a store selling reading spectacles for US $1 was located next to the IDES office, this distribution of responses most likely reflects a lack of knowledge about the condition and its readily accessible treatment. Many people who require near vision correction can benefit from ready-made or ‘over-the-counter’ reading spectacles. These individuals should, however, undergo a full eye examination to check for other pathology or the effects of systemic conditions such as diabetes or high blood pressure.

**Table:** Table 1. Pereceived barriers and benefits to correcting near vsion

	Percentage reporting perceived degree of impact (%)			
	Greatly	Moderately	Some	Not at all
**Barrier^a^**				
Cost of reading spectacles (n=130*)	49.0	8.0	11.5	31.5
Employed (n=37)	46.0	2.7	18.9	32.4
Unemployed (n=93)	50.5	9.7	8.6	31.2
Availability of an eye doctor (n=131*)	48.0	6.0	9.0	37.0
Employed (n=37)	37.8	10.8	16.2	35.1
Unemployed (n=94)	52.1	4.3	6.4	37.2
**Benefit^b^**				
Reading print (n=133)	82.7	10.5	5.3	1.5
Employed (n=37)	86.5	5.4	5.4	2.7
Unemployed (n=96)	81.3	12.5	5.2	1.0
Preparing meals, using hand tools, or sewing	47.4	15.0	12.0	25.6
Employed (n=37)	64.9	13.5	5.4	16.2
Unemployed (n=96)	40.6	15.6	14.6	29.2
Grooming, shaving or applying makeup (n=133)	30.8	10.5	10.6	48.1
Employed (n=37)	45.9	10.8	5.4	37.8
Unemployed (n=96)	25.0	10.4	12.5	52.1
^a^ Degree to which each barrier was perceived to prevent correction of near vision. ^b^ Degree of Impact of reading spectacles on selected tasks.
^*^ Three Individuals from the overall study population did not provide answers to the cost barrier question and two Individuals did not provide an answer to the question of availability of an eye doctor as a barrier
Note: due to rounding, some rows may not add up to 100%.

Over two-thirds of the respondents (68 percent) had been examined by an eye care professional at some point in their lives, but only 30 per cent (n=40) had been examined in the previous year. Our population was predominantly Hispanic and African American. Considering that the rates of blindness and visual impairment in these groups is significantly greater than that of non-Hispanic whites,^3^ this amounts to suboptimal use of eye care services relative to the recommended guidelines for high-risk groups from the American Academy of Ophthalmology.

Employment status correlated with the frequency with which patients accessed an eye care professional. Whereas almost 80 per cent of respondents who were employed had seen an eye doctor, less than 64 per cent of respondents who were unemployed had similarly accessed care (p<0.05).

In a country where services are readily available and spectacles are affordable, nearly half of the participants were not aware that this was case. All of the participants would have benefited from a pair of reading spectacles.

Our study is the first to examine how presbyopia affects a resource-poor population in the USA. The survey highlights the need for continued efforts to ensure that economically disadvantaged and minority populations gain access to eye care services and utilise them. Practical solutions include outreach activities and education of health care providers in low-resource communities about the ease with which their patients may remedy their prebyopic needs with inexpensive, over-the-counter, ready-made reading spectacles. Health care providers should also be educated about the increased rates of blindness and visual impairment in the African American and Hispanic populations so that they can encourage and refer individuals in these population groups for regularly scheduled eye examinations.
